# Grade 4 Mucositis Secondary to Methotrexate Use in a Pediatric Patient With Stage IV Burkitt Lymphoma: A Case Report

**DOI:** 10.7759/cureus.90944

**Published:** 2025-08-25

**Authors:** Fadia Y Abdala Mendoza, Circe Ancona Castro

**Affiliations:** 1 Pediatrics, Instituto de Seguridad y Servicios Sociales de los Trabajadores del Estado (ISSSTE) Regional Hospital, Monterrey, MEX; 2 Dermatology, Instituto de Seguridad y Servicios Sociales de los Trabajadores del Estado (ISSSTE) Regional Hospital, Monterrey, MEX

**Keywords:** cd20-negative burkitt lymphoma, high-dose methotrexate, methotrexate toxicity, pediatric mucositis, severe mucositis

## Abstract

Non-Hodgkin lymphoma (NHL) is a monoclonal neoplastic proliferation of lymphoid cells that can arise in different components of the immune system.

Methotrexate (MTX), an antifolate agent, is widely used in the treatment of malignancies and autoimmune diseases. One of its most significant toxicities is gastrointestinal mucositis, a dose-dependent mucosal injury that can involve the entire alimentary tract and manifest as painful oral and intestinal ulcerations.

We present the case of a pediatric patient with stage IV Burkitt lymphoma who developed severe mucocutaneous toxicity after MTX administration. This report also reviews risk factors associated with heightened toxicity and highlights strategies for prevention and management of MTX-induced oral mucositis, with particular emphasis on the clinical importance of delayed drug clearance, which increases the risk of multisystem toxicity and mortality.

## Introduction

Non-Hodgkin lymphoma (NHL) is a monoclonal neoplastic proliferation of lymphoid cells that can arise in multiple sites within the immune system, including lymph nodes, bone marrow, spleen, liver, and gastrointestinal tract. Burkitt lymphoma, a subtype, typically presents as bulky abdominal disease, often involving the ileocecal region [1,2].

Methotrexate (MTX), an antifolate chemotherapeutic agent, is used in the treatment of both neoplastic and autoimmune disorders. High-dose MTX (HDMTX), defined as >500 mg/m² IV infusion, may precipitate within renal tubules, and its metabolites can cause acute kidney injury (AKI). Delayed renal clearance prolongs MTX exposure and increases the risk of systemic toxicity [3].

Gastrointestinal mucositis is one of the major side effects of chemotherapy, resulting from damage to the mucosal lining from the mouth to the anus, with both oral and intestinal involvement [4]. Oral mucositis occurs in about 20% of pediatric patients receiving HDMTX for acute lymphoblastic leukemia (ALL), despite current preventive strategies. Although pharmacokinetically guided, risk-adapted dosing has reduced its incidence, significant interpatient variability persists even at comparable 24-hour MTX levels [5].

While chemotherapy-induced mucositis typically affects non-keratinized oral mucosa, diffuse skin and mucosal involvement is rarely reported in children. Unlike the more common presentation of isolated oral ulcers, our patient developed extensive erosions and blistering across multiple body sites.

We present the case of a pediatric patient with stage IV Burkitt lymphoma and multiple predisposing risk factors, who developed severe mucocutaneous toxicity secondary to MTX chemotherapy. This report highlights risk factors contributing to MTX toxicity and outlines strategies for prevention and management, with particular emphasis on the dangers of delayed drug clearance and the risk of systemic toxicity and death.

## Case presentation

An 11-year-old male with a history of AKI and miliary tuberculosis was recently diagnosed with stage IV Burkitt lymphoma. Immunohistochemistry showed CD20 (+++), CD10 (+), C-Myc (+++), and negative CD3. Chemotherapy was initiated.

After the first cycle (ifosfamide 800 mg/m²/day with MESNA, vincristine 1.5 mg/m²/day, MTX 5 g/m²/day, cytarabine 150 mg/m²/day ×2 doses, etoposide 100 mg/m²/day ×2 doses, and folinic acid 15 mg/m² every six hours), the patient developed right pleural effusion, ascites, intrinsic renal injury, and mucositis.

**Figure 1:**
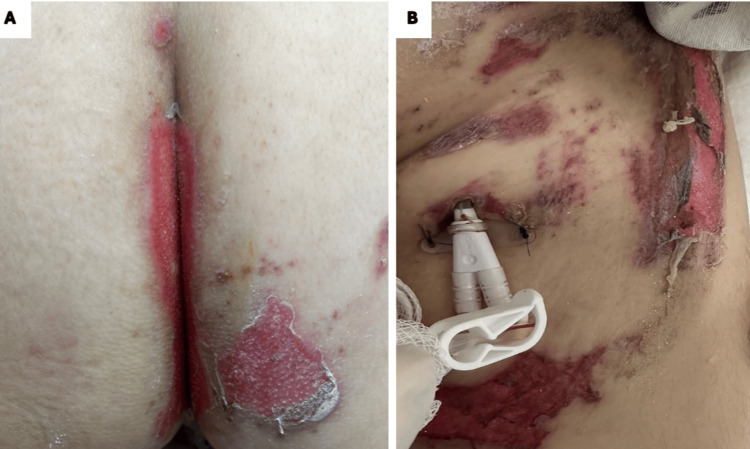
Perianal and inguinal mucositis lesions Multiple ulcerations and ruptured blisters with residual blister roofs in the perianal (A) and inguinal (B) areas.

On examination, there was disseminated dermatosis involving the scalp, oral mucosa (upper and lower lips, oral commissures), and trunk (anterior thorax, mesogastrium, hypogastrium, pubic region, and gluteal/perianal areas). The genital region (prepuce) and inner thighs were also affected. Lesions appeared as multiple erosions and ruptured blisters with residual roofs, surrounded by mild erythema, measuring 2-10 cm in diameter, predominantly in the perianal (Figure 1A) and inguinal (Figure 1B) regions.

**Figure 2:**
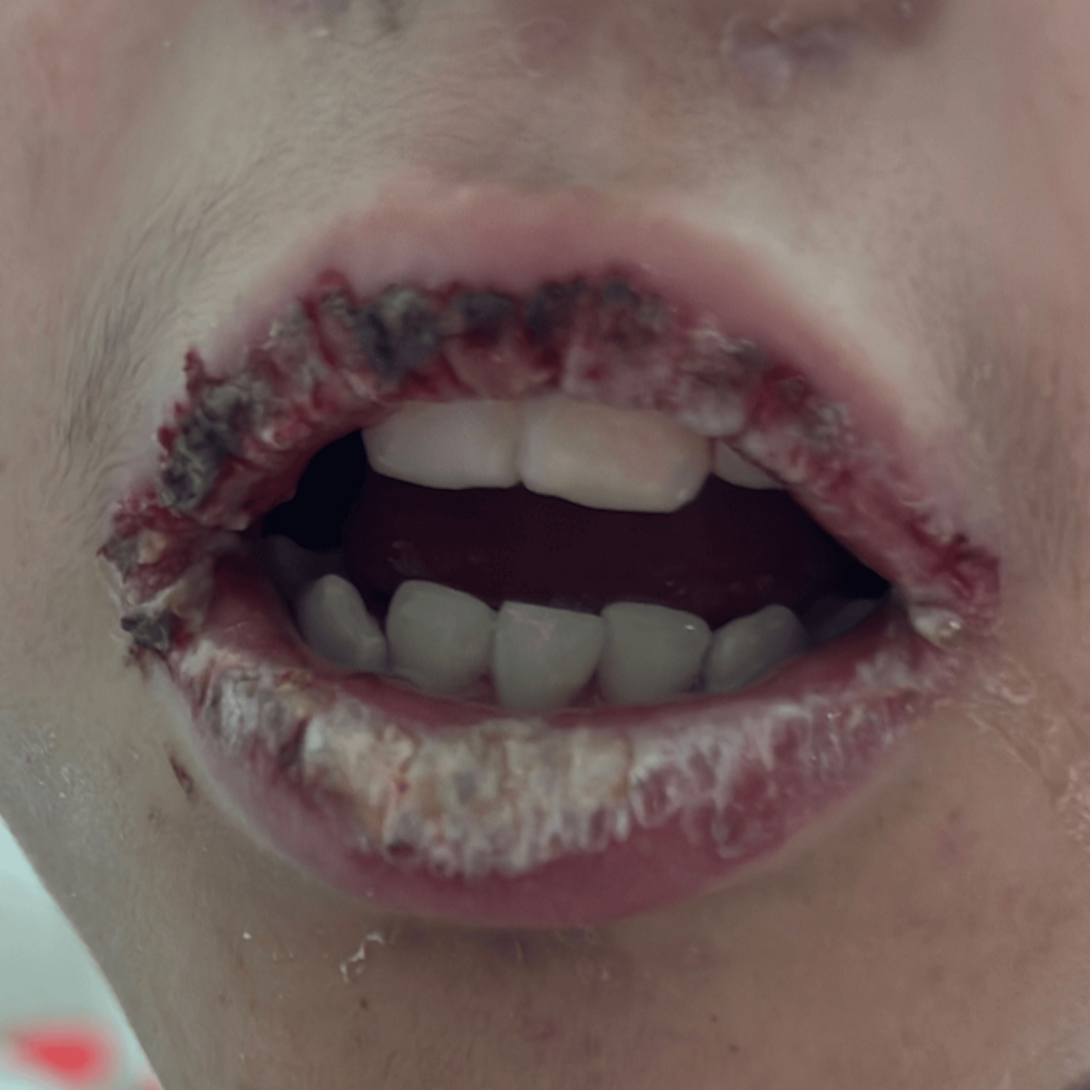
Oral mucosa erosions with serous and hemorrhagic crusts

The oral mucosa demonstrated erosions covered with hemorrhagic and serous crusts (Figure 2).

**Figure 3:**
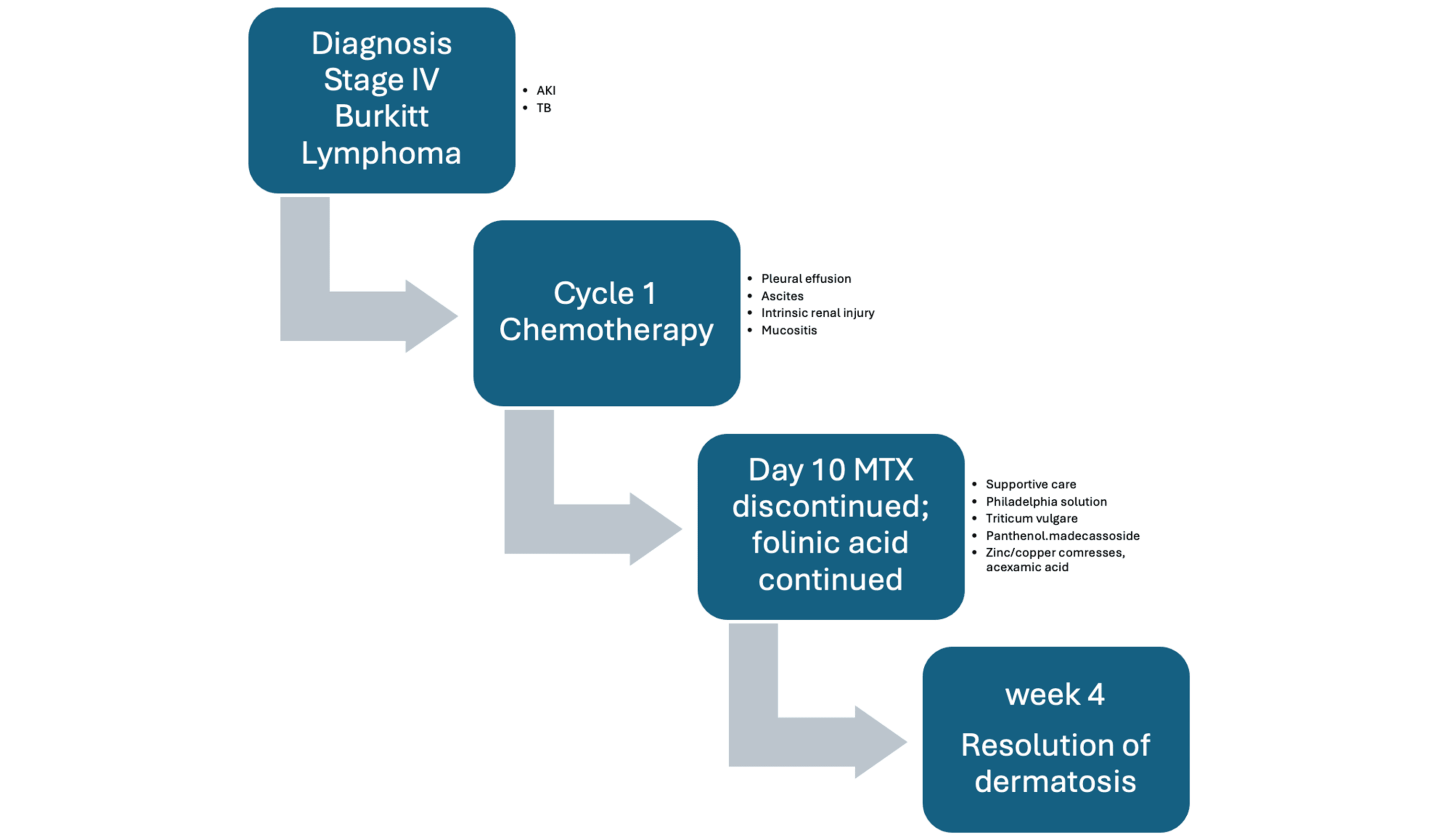
Timeline of case chronology Image Credit: Authors' creation.

Given the grade 4 mucocutaneous toxicity, MTX was discontinued while folinic acid rescue was maintained. Supportive topical management included Philadelphia solution mouthwash (lidocaine 2%, magnesium-aluminum gel, diphenhydramine), *Triticum vulgare* every 12 hours, 5% panthenol with madecassoside every 12 hours, zinc/copper sulfate and camphor compresses every 12 hours, and acexamic acid every 12 hours (Figure 3).

**Figure 4:**
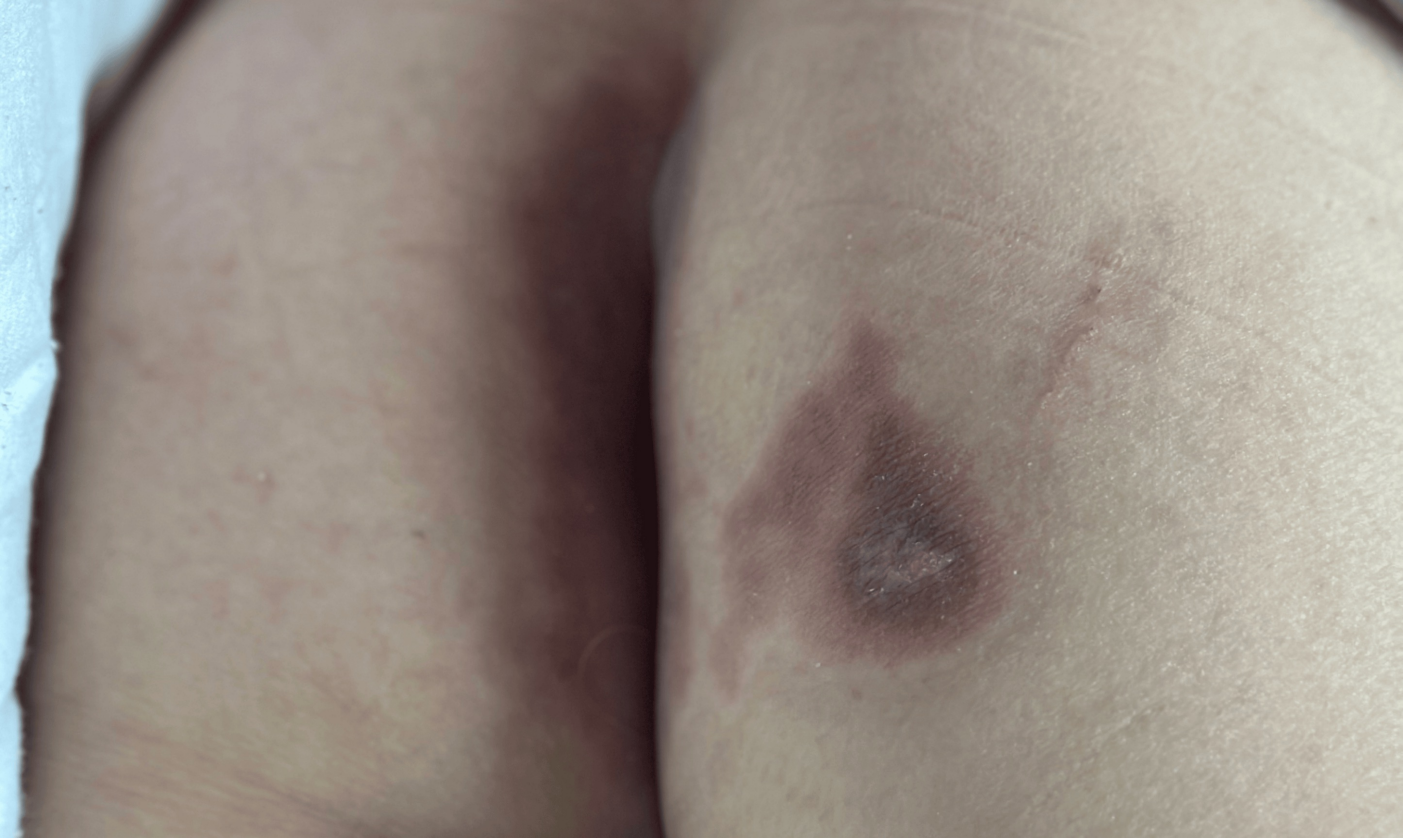
Resolution of dermatosis in the perianal area

The patient showed progressive improvement with complete resolution of dermatosis within one month (Figure 4).

Written informed consent was obtained from the patient’s legal guardian for publication of this case report and the accompanying images.

## Discussion

In this patient, mucositis presented as diffuse mucocutaneous involvement, extending beyond the typical non-keratinized oral mucosa to include the scalp, trunk, perianal, and genital regions. While chemotherapy-induced mucositis typically develops within 5-14 days of treatment and affects 30%-40% of patients, widespread skin and mucosal erosions are rare in pediatric cases [5,6] (Table 1).

**Table 1:** Differential diagnosis HSV: herpes simplex virus, CMV: cytomegalovirus, HSCT: hematopoietic stem cell transplantation. Source: [7-11].

Differential diagnosis	Key features/clues
Infectious stomatitis (HSV, CMV, *Candida*)	Vesicular or ulcerative lesions, fever, positive viral culture or PCR, fungal scrapings [7]
Stevens-Johnson syndrome/toxic epidermal necrolysis	Widespread mucosal involvement with skin detachment, prodromal fever, systemic symptoms [8]
Graft-versus-host disease (GVHD)	Occurs post-HSCT; lichenoid mucosal changes, often chronic [9]
Aphthous ulcers/Behçet's disease	Recurrent oral and genital ulcers, systemic vasculitis findings [10]
Nutritional deficiencies (e.g., B12, folate, iron)	Atrophic glossitis, angular cheilitis, anemia [11]

The pathogenesis of mucositis involves five sequential, overlapping stages-(1) initiation: chemotherapy or radiation causes DNA and cellular injury, triggering the production of reactive oxygen species (ROS); (2) upregulation: ROS and cellular damage stimulate proinflammatory cytokine release; (3) signal amplification: cytokines perpetuate tissue damage through positive feedback loops; (4) ulceration: painful lesions prone to bacterial colonization develop, further increasing tissue damage and inflammation; and (5) healing: epithelial proliferation, cellular differentiation, and tissue restoration occur [12].

Oral complications are classified as either acute or late. Acute complications arise during therapy and include oropharyngeal mucositis, xerostomia, sialadenitis, fungal or viral infections, and taste alterations. Late complications may present as fibrosis, mucosal atrophy, xerostomia, necrosis, dysgeusia, or dysphagia [12,13].

MTX is an antimetabolite that inhibits dihydrofolate reductase, a key enzyme in folate metabolism, thereby impairing DNA and protein synthesis. By blocking tetrahydrofolate production, MTX reduces cellular proliferation capacity and induces apoptosis. Its adverse effects are dose- and frequency-dependent, with greater severity seen at antineoplastic doses [14] (Table 2).

**Table 2:** Risk factors for methotrexate toxicity Source: [14].

Risk factors
Overdose due to dosing error (daily instead of weekly)
Presence of infection	
Folic acid deficiency	
Hypoalbuminemia	
Renal insufficiency	
Third space formation: ascites or pleural effusion	
Alcohol intake	
Advanced age	
Recent initiation of methotrexate (MTX) or dose increase	
Drugs:	
By decreased renal elimination: aminoglycosides, cyclosporine, nonsteroidal anti-inflammatory drugs (NSAIDs), sulfonamides, probenecid, salicylates, penicillin, colchicine, cisplatin, proton pump inhibitors	
By displacement from plasma protein binding: salicylates, probenecid, sulfonamides, barbiturates, phenytoin, retinoids, sulfonylureas, tetracyclines, diuretics	
By synergistic toxicity, inhibiting dihydrofolate reductase (DHFR): trimethoprim/sulfamethoxazole	

In chronic low-dose MTX therapy, folic acid supplementation is recommended; in high-dose therapy, leucovorin is preferred to mitigate toxicity [15].

Mucocutaneous reactions, including mucosal ulcers, psoriatic erosions, alopecia, photosensitivity, acral erythema, erythema multiforme, urticaria, and vasculitis, are common in acute toxicity, particularly in the absence of folate supplementation. The risk of adverse effects is highest in the first six months of treatment. Long-term monitoring should include renal and hepatic function tests (given that MTX is primarily renally excreted), as well as complete blood counts, with mean corpuscular volume (MCV) serving as an early marker of toxicity. Systemic complications may include myelosuppression, mucositis, hepatotoxicity, neurotoxicity, and pulmonary toxicity [15].

A retrospective study at the Hospital Clínic de Barcelona suggested that oral ulcers may be early indicators of MTX-induced bone marrow toxicity. Five patients developed oral mucositis after MTX administration. Reported risk factors included NSAID use, dosing errors, infections, and folate deficiency. Drug clearance was slower in lymphoma cases compared with sarcoma cases, underscoring the need for individualized dosing. Ascites and pleural effusion should also be ruled out before initiating high-dose MTX (HDMTX) regimens [15].

The presence of third-space fluid during leucovorin rescue and impaired renal clearance in AKI further increases the risk of mucocutaneous toxicity, highlighting the importance of careful monitoring and individualized management [16].

In this patient, several factors likely prolonged MTX exposure and amplified toxicity. First, a history of AKI compromised renal tubular clearance, the primary elimination pathway for MTX, leading to delayed elimination and elevated systemic levels [17]. Second, the presence of ascites and a right pleural effusion created “third-space” compartments where MTX could sequester and slowly re-equilibrate, extending its effective half-life and tissue exposure. These factors contributed to unusually extensive mucocutaneous toxicity, with erosions involving the scalp, trunk, perianal, and genital regions, well beyond the typical oral distribution of chemotherapy-induced mucositis. The severity and distribution required discontinuation of MTX and intensive supportive care, underscoring the importance of early recognition in pediatric patients.

While pediatric HDMTX commonly causes oral mucositis, diffuse mucocutaneous involvement, including perianal/genital and widespread cutaneous erosions, is rare. Compared with the usual presentation limited to oral mucosa, this case is notable for its grade 4 severity and broad distribution, closely associated with HDMTX administration and improved following MTX cessation and optimized rescue therapy [18].

## Conclusions

MTX remains a cornerstone of pediatric oncology, yet its use carries significant risk when clearance is delayed. This case highlights the importance of assessing renal function before HDMTX administration, maintaining aggressive hydration, and promptly adjusting leucovorin rescue when delayed clearance is suspected. Early recognition of severe mucocutaneous toxicity and rapid modification of therapy are crucial to prevent life-threatening complications. Pediatric-specific data on MTX pharmacokinetics remain limited, emphasizing the need for individualized dosing, close monitoring of clinical and laboratory markers, and multidisciplinary collaboration. Implementing evidence-based protocols that account for patient-related risk factors, such as renal impairment, hypoalbuminemia, or folate deficiency, can help minimize toxicity and optimize outcomes.

In pediatric patients receiving HD-MTX, strict pre-treatment renal evaluation, proactive hydration, and timely leucovorin adjustments are essential for safe and effective therapy.
